# Refining caregiver vulnerability for clinical practice: determinants of self-rated health in spousal dementia caregivers

**DOI:** 10.1186/s12877-019-1033-2

**Published:** 2019-01-22

**Authors:** Roland von Känel, Brent T. Mausbach, Joel E. Dimsdale, Michael G. Ziegler, Paul J. Mills, Matthew A. Allison, Thomas L. Patterson, Sonia Ancoli-Israel, Igor Grant

**Affiliations:** 1 Department of Consultation-Liaison Psychiatry and Psychosomatic Medicine, University Hospital Zurich, University of Zurich, Culmannstrasse 8, CH-8091 Zurich, Switzerland; 2 Department of Psychiatry, University of California San Diego, La Jolla, California USA; 3 Department of Medicine, University of California San Diego, La Jolla, California USA; 4 Department of Family Medicine and Public Health, University of California San Diego, La Jolla, California USA

**Keywords:** Clinical management, Dementia caregivers, Elderly people, Health risk, Psychological stress, Quality of life, Self-rated health

## Abstract

**Background:**

Caregivers of a family member with a chronic disability or illness such as dementia are at increased risk for chronic disease. There are many factors that contribute to dementia caregiver vulnerability and these factors can be challenging to assess in clinical settings. Self-rated health (SRH) is an independent measure of survival and physical health in the elderly. As an inclusive measure of health, SRH has been proposed as a reliable way to assess a patient’s general health in primary care. Therefore, we sought to identify determinants of poor/fair SRH versus categories of at least good SRH in informal caregivers.

**Methods:**

In a cross-sectional study, we examined 134 elderly (≥55 years) providing in-home care for a spouse with dementia who rated their own health with a single-item question: “In general, would you say your health is excellent, very good, good, fair or poor?”. In a multivariable model, we compared caregivers with poor/fair SRH to those with good, very good, or excellent SRH on demographics, health characteristics (health behaviors, physical health indicators, psychosocial factors) and caregiving-specific stress (a composite index/total of four caregiving-specific stressors: years of caregiving, dementia severity, care recipient functional impairment and perceived caregiver burden).

**Results:**

Compared with caregivers who rated their own health as either good (31.3%), very good (38.8%) or excellent (14.2%), caregivers with poor/fair SRH (15.7%) were more likely to have lower physical function and total greater caregiving-specific stress. More years of caregiving, severe dementia and care recipient functional impairment, but not perceived caregiver burden, were also more likely among caregivers with poor/fair SRH. Additionally, high negative affect and low positive affect were more likely in caregivers with poor/fair vs. good or excellent and very good or excellent SRH, respectively.

**Conclusions:**

Caregivers with poor/fair SRH were characterized by higher levels of medical comorbidity, low physical function, high negative, but low positive affect and longer duration of caregiving, as well as more severe dementia and greater functional impairment of the care recipient. These findings suggest that caregivers need to be more closely evaluated and targeted for preventive interventions in clinical practice.

**Trial Registration:**

ClinicalTrials.gov registration number: NCT02317523.

**Electronic supplementary material:**

The online version of this article (10.1186/s12877-019-1033-2) contains supplementary material, which is available to authorized users.

## Background

Caregiving for a family member with a chronic disability or illness has been associated with increased mortality risk [1, 2], as well as adverse psychologic, cognitive, and physical outcomes [3–5], including incident cardiovascular disease [6–8]. Caregivers are more stressed than non-caregivers [9], and negative effects of caregiving on both physical health and mortality are more commonly found in psychologically distressed caregivers and those facing dementia-related stressors [1, 7, 10–12].

There are numerous risk factors of adverse health outcomes in caregivers. These include demographic factors, health and physical function, social support and type of caregiving, with a particularly high risk in dementia vs. non-dementia caregivers [13]. Yet, the predictive ability of these risk factors should be viewed cautiously, as they are often intercorrelated and have not been simultaneously examined [13].

Vulnerable caregivers at high risk of adverse health outcomes should be identified [4, 14, 15]. However, this task is challenging in clinical settings, where short consultations, reimbursement structure and lack of training limit extensive history taking, risk assessments [16], and the implementation of practice guidelines [17]. To overcome this shortcoming, it has been suggested that health professionals should simply ask caregivers about how they perceive their own health, as those with poor self-rated health (SRH) are at an increased risk of serious medical complications and mortality [15, 16, 18]. In this regard, population-based studies show a gradual increase in mortality risk, independent of covariates across response categories of excellent, very good, good, fair and poor SRH, with twice the risk in individuals with poor vs. excellent SRH [19].

In adults 60 years and older, fair or poor vs. at least good SRH is a powerful predictor of all-cause, cardiovascular and cancer mortality such that SRH may also be a useful tool for a quick and simple identification of elders in need for more intensive clinical evaluation [20]. Since SRH is associated with comorbid illness, health behaviors, functional status, psychological distress, social support and demographic factors, assessments of SRH with a single-item question may yield an inclusive measure of health [21]. As such, SRH has been proposed for disease screening [21] and as a reliable way to assess patients’ general health situation in primary care [22].

About 30% of caregivers rate their own health as fair or poor [23], and caregivers have significantly poorer SRH than non-caregivers, even when adjusting for demographic factors, social support and long-term illness [24]. In dementia caregivers, caregiver burden was found to be the strongest predictor of poor SRH [25] and, in a vicious cycle, the poor SRH accentuated caregiver distress [26]. However, the determinants of poor/fair SRH versus each category of better SRH (i.e., versus good, very good and excellent SRH) have not systematically been investigated in dementia caregivers [23].

On the whole, SRH provides a summary statement about numerous aspects of subjective and objective health within the perceptual framework of an individual patient [21], and the process may also apply to dementia caregivers in clinical settings. SRH can be recommended as an easy-to-administer routine health indicator that can be used as a screening tool by clinicians, while at the same time allowing caregivers to generate a genuine perspective of their own health [21]. Moreover, identification of some of the above delineated determinants of SRH in caregivers with poor/fair SRH could help to make decisions about further clinical evaluation and target interventions in the group of vulnerable caregivers with the greatest health risk.

Therefore, we sought to identify demographic factors (i.e. age, sex, education), health characteristics (i.e., health behaviors, physical health indicators, psychosocial factors) and caregiving-specific stressors (i.e., years of caregiving, dementia severity, functional impairment of the care recipient, perceived caregiver burden) that may differentiate dementia caregivers with poor/fair SRH from those with good, very good, or excellent SRH. Based on the existing literature, all of these determinants of SRH could be hypothesized to differentiate caregivers with different categories of SRH. For the purpose of this study, we were particularly interested in potentially modifiable components of SRH as potential targets to improve clinical care in dementia caregivers. We specifically hypothesized that caregivers with poor/fair SRH are at an increased risk of poor physical health, high negative/low positive affect, and of experiencing caregiving-specific stress.

## Methods

### Study participants and design

The participants of this cross-sectional study comprised 134 of 136 caregivers who were enrolled between 2/2015 and 11/ 2017 in the University of California, San Diego (UCSD) Alzheimer’s Caregiver Study for a randomized controlled trial aimed at improving caregiver psychobiological health (ClinicalTrials.gov registration number: NCT02317523). The intervention has been described in detail elsewhere [27]. In brief, six face-to-face sessions were conducted in caregivers’ homes. Intervention 1 is a behavioral activation intervention targeting engagement in pleasant leisure activities. Intervention 2 (comparison) is a support intervention but teaches skills like managing problem behaviors and improving communication. Data reported here are from the baseline visit, obtained before randomization. The baseline visit consisted of a series of questionnaires, administered by interview by a trained research associate. The interview took approximately 1.5–2 h in duration and the participants were paid $100 for completing the assessment.

Our study used community sampling strategies to recruit participants. We presented the study at various community support groups, health seminars for elderly (generally) and caregivers (specifically), and also received referrals from community providers who serve caregivers and dementia patients (e.g., professionals at adult day care centers; respite providers). The majority of subjects were recruited through community agencies serving active Alzheimer’s disease patients and their caregivers. These included Alzheimer’s organizations (e.g., Alzheimer’s association) and a variety of caregiver support groups, churches, health fairs, and day care facilities. Additional recruitment of a minor fraction of participants was through the UCSD Alzheimer’s Disease Research Center. We screened 248 participants for eligibility, of which 150 met our inclusion criteria. Of these, 136 were willing to participant and provided consent. Two participants were excluded from the present analysis due to missing demographic and SRH data, yielding a final sample of 134.

To be eligible, caregivers had to be 55 years or older, English-speaking, provide at least 20 h per week of in-home care for a spouse with dementia and endorse at least mild depressive symptoms (i.e., mild level of distress) as per a score of ≥5 on the Patient Health Questionnaire-9 [28] at the time of enrollment. A specific diagnosis of Alzheimer’s disease, as opposed to other forms of dementia, was not required as an inclusion criterion.

Exclusion criteria were current treatment for any malignancies, severe chronic illnesses requiring ongoing medical treatment (e.g., chronic obstructive pulmonary disease, renal failure), severe hypertension (> 200/120 mmHg), major psychiatric illnesses (e.g., schizophrenia, bipolar disorder), prior or ongoing participation in a behavioral caregiver intervention, or treatment with steroids, non-selective beta-blocking, or anticoagulant medications.

### Measures

#### Self-rated health

Self-rated health was assessed with the single-item question, “In general, would you say your health is:” with the response categories of “excellent”, “very good”, “good”, “fair” and “poor”. The item was presented as part of the 12-Item Short-Form Health Survey [29] and corresponds to the most widely used response option for the evaluation of one’s own health status in the US [21]. The measure of SRH used in our study showed moderate test-retest reliability (kappa coefficient = 0.41) in a nationally representative sample of US adults 60–80 years [30]. As discussed elsewhere, there is no clear criterion for the predictive validity of SRH because there exists no direct objective gold standard measure of “true health” [21]. Moreover, the usefulness of self-ratings of health as valid measures to predict objective health indicators depends upon the clinical, cultural, and research context [21].

#### Determinants of self-rated health analyzed

We assessed demographics factors (age, sex, educational level), health characteristics (health behaviors, physical health indicators, psychosocial factors) and caregiving-specific stress (objective and subjective caregiving-specific stressors). Health behaviors were body mass index (BMI), physical activity, smoking, and alcohol consumption. Physical health indicators were physical health problems and caregiver physical function. Psychosocial factors included positive and negative affect and social support. Objective caregiving-specific stressors were total years of caregiving, dementia severity and care recipient functional impairment; perceived caregiver burden was used as a subjective caregiving-specific stressor.

#### Demographic factors

A research associate administered interview-based demographic questions and recorded the responses with respect to age, sex and educational level as a measure of socioeconomic status; the latter was categorized into higher education (i.e., some college/associate degree, college graduate, master’s/other post-graduate degree, doctoral degree) vs. lower education (i.e., grade 12/high school diploma/general educational development, vocational/training school). Only two participants indicated less than grade 12; these were lumped with caregivers in the lower education group.

#### Health behaviors

The BMI was calculated in kg/m^2^ based on weight and height; both measured by research associates. The Rapid Assessment of Physical Activity scale was used to assess the amount of light (e.g. stretching, vacuuming), moderate (e.g. fast walking, strength training), and strenuous (e.g. jogging, stair machine) physical activities in a typical week (total score 0–6; 6 indicates the greatest amount of physical activity) [31]. Smoking status was categorized into ever (i.e., all former plus 5 current smokers) vs. never smoking. The amount of consumed alcohol was scored on an ordinal scale based on the number of days on which caregivers had at least one alcoholic drink in the past month (scores ranges from 0 “0 days” to 6= “all 30 days”).

#### Physical health indicators

A review of systems and a health history questionnaire were used, interview-style, to assess physical health problems by asking caregivers the question “Do you currently have, or has a doctor ever told you that you have, any of the following health problems (heart attack, stroke, high blood pressure, heart disease, diabetes, high cholesterol, lung disease, liver disease, kidney problems, sleep apnea, cancer, thyroid disease)?” The number of positive items was summed to reflect medical comorbidity (total score 0–12). Caregiver physical function was assessed with 5 items of the 12-Item Short-Form Health Survey physical summary scale: limitations in moderate activities (score ranges from 1= “limited a lot” to 3= “not limited at all”) and climbing several flights/stairs (score ranges from 1 = “limited a lot” to 3 = “not limited at all”); limitations in accomplishments (score ranges from 1= “all of the time” to 5= “none of the time”) and in kind of work or other activities (score ranges from 1 = “all of the time” to 5 = “none of the time”) due to physical health, and pain interfering with caregivers’ normal work (score ranges from 1 = “extremely” to 5= “not at all”) during the past 4 weeks [29]. Because of the different response formats, standardized z-scores of each item were summed and the total divided by 5 to obtain a physical function total score (range of the attainable score between − 2.25 and 0.86; higher scores indicate better physical function).

#### Psychosocial factors

Positive and negative affect in the past few weeks was assessed with the Positive and Negative Affect Scale [32], which has shown its validity in older adults [33]. Caregivers rated 10 items for negative affect (e.g., upset, afraid) and positive affect (e.g., excited, active) each on a 5-point Likert scale (1= “very slightly or not at all”, 5 = “extremely”; total score 10–50 for either scale). Cronbach’s *α* was 0.85 for the negative affect scale and 0.87 for the positive affect scale indicating good reliability in our sample. Social support was assessed with the Enhancing Recovery in Coronary Heart Disease Social Support Inventory, by asking about how often emotional support (4 items), practical support (1 item) and informational support (1 item) was available from any network member [34]. Example items were: “Is there someone available to you who shows you love and affection” and “Is there someone available to help with daily chores?” Each item was rated on a 5-point Likert scale (0= “none of the time”, 4= “all the time”; total score 0–24). Cronbach’s *α* was 0.83 for the total scale indicating good reliability.

#### Caregiving-specific stressors

Objective measures of caregiving-specific stress were the number of years of caregiving, asked by the research assistant, and care recipients’ dementia severity and functional impairment. Dementia severity was assessed with the Clinical Dementia Rating scale which incorporates 6 different behavioral and cognitive domains including memory, orientation, judgment/problem solving, community affairs, home/hobbies, and personal care [35]. Each domain was evaluated separately with scores ranging from 0 (non-demented) to 3 (severely demented). An overall dementia rating (total score 0–3) was then computed from all domain scores. The Clinical Dementia Rating scale has been shown to have both high interrater reliability and validity [36]. Functional impairment of care recipients was assessed with the Activities of Daily Living Questionnaire for patients with dementia, which covers 6 different areas referring to self-care, household, employment, shopping, travel, and communication. Total scores (range between 0 and 100%) were expressed as percent impairment in performing activities of daily living (0–33% = no or mild impairment, 34–66% = moderate impairment, 67–100% = severe impairment) [37].

As a subjective measure of caregiving-specific stress, perceived caregiver burden was assessed with the 12-item short form of the Zarit Burden Interview [38]. Item examples were “Do you feel that because of the time you spend with (care recipient) that you don’t have enough time for yourself?” and “Do you feel you have lost control of your life since (care recipient’s) illness?” Each item is rated on a 5-point Likert scale (1 = “never”, 5 = “nearly always”; total score 12–60). Cronbach’s *α* was 0.81 for the total scale indicating good reliability in our sample.

A primary caregiving-specific stress measure “caregiving-specific stress total” (range of the attainable score between − 1.91 and 3.14) was formed from averaging standardized z-scores of the sum scores of the four caregiving-specific stressors (years of caregiving, dementia severity, care recipient functional impairment, perceived caregiver burden).

### Data analysis

Data were analyzed using SPSS 23.0 for Windows (SPSS Inc., Chicago, IL) with level of significance at *p* < 0.05. Missing values for BMI (4 cases), affect (2 cases) and the Clinician Dementia Rating Scale (1 case) were replaced with the expectation maximization algorithm. Analysis of variance and Pearson chi-square test (Fisher’s Exact test where appropriate) were used to compare caregiver groups with a different SRH status on demographic factors, health characteristics (health behaviors, physical health indicators, psychosocial factors), and caregiving-specific stress (caregiving-specific stress total and individual caregiving-specific stressors). Pearson correlation analysis was used to estimate the univariate relationship between two variables.

Multinomial logistic regression analysis was applied to determine the crude and fully-adjusted probability of predictors of poor/fair SRH as the reference category vs. the categories of 1) good, 2) very good, or 3) excellent SRH. To prevent model overfitting, a maximum of 13 covariates was allowed. Components of “health” in self-assessments were selected as potential predictors of SRH based on the literature (age, sex, education, BMI, physical activity, smoking status, alcohol consumption, physical health problems, caregiver physical function, negative affect, positive affect, social support [21]; caregiving-specific stressors [25, 26]) and entered in one block. We then performed post hoc analyses whereby replacing the variable “caregiving-specific stress total” by individual caregiving-specific stressors (i.e., years of caregiving, clinical dementia rating, care recipient functional impairment, perceived caregiver burden) in four separate models. Model output indicated no concern for multicollinearity.

## Results

### Participant characteristics

A total of 21 (15.7%) caregivers reported “poor/fair SRH” (3 poor and 18 fair SRH). Most of the caregivers reported either good (*n* = 42, 31.3%), very good (*n* = 52, 38.8%) or excellent (*n* = 19, 14.2%) SRH. There were no differences in demographic factors and measures of caregiving-specific stress between the different SRH groups (see Table 1).

**Table 1:** Characteristics of 134 Alzheimer caregivers per categories of self-rated health (SRH) with significant group differences

	Attainable score of scales	All (*n* = 134)	Poor/fair SRH (*n* = 21)	Good SRH (*n* = 42)	Very good SRH (*n* = 52)	Excellent SRH (*n* = 19)	*P* (between groups)
*Demographic factors*							
Age, years, mean (SD)		74.1 (8.3)	73.9 (9.1)	72.7 (8.4)	75.5 (7.9)	73.8 (7.8)	0.427
Sex, female, *n* (%)		105 (78.4)	18 (85.7)	33 (78.6)	39 (75.0)	15 (78.9)	0.823
Higher education, *n* (%)		104 (77.6)	15 (71.4)	31(73.8)	40 (76.9)	18 (94.7)	0.226
*Health behaviors*							
Body mass index, kg/m^2^, mean (SD)		26.1 (5.5)	28.1 (8.1) ^1)^	27.1 (4.7) ^4)^	25.5 (4.9)	23.5 (4.2)	0.026
Physical activity, score, mean (SD)	0 to 6	3.31 (1.62)	2.71 (1.35) ^1) 2)^	2.76 (1.28) ^4) 5)^	3.62 (1.71)	4.37 (1.71)	< 0.001
Ever smoking, *n* (%)		58 (43.3)	11 (52.4)	16 (38.1)	20 (38.5)	11 (57.9)	0.346
Alcohol consumption, score, mean (SD)	0 to 6	2.03 (2.26)	1.10 (2.02) ^1) 2)^	1.50 (1.88) ^4) 5)^	2.50 (2.36)	2.95 (2.46)	0.009
*Physical health indicators*							
Physical health problems, score, mean (SD)	0 to 12	1.93 (1.54)	3.00 (1.87) ^1) 2) 3)^	2.21 (1.54) ^4) 5)^	1.48 (1.29)	1.37 (1.01)	< 0.001
Caregiver physical function, z-score, mean (SD)	−2.25 to 0.82	0 (0.81)	−0.84 (0.79) ^1) 2) 3)^	−0.10 (0.71) ^4) 5)^	0.25 (0.72)	0.48 (0.56)	< 0.001
*Psychosocial factors*							
Negative affect, score, mean (SD)	10 to 50	21.0 (6.8)	26.4 (8.5) ^1) 2) 3)^	19.3 (5.5)	21.3 (6.3) ^6)^	17.8 (5.4)	< 0.001
Positive affect, score, mean (SD)	10 to 50	32.4 (7.0)	29.7 (6.1) ^1)^	30.7 (6.7) ^4)^	32.7 (6.6) ^6)^	37.9 (6.9)	< 0.001
Social support, score, mean (SD)	0 to 24	13.7 (5.4)	12.7 (6.8)	14.9 (4.9)	12.8 (5.1)	14.6 (5.1)	0.183
*Caregiving-specific stressors*							
Years of caregiving, mean (SD)		4.59 (3.54)	5.71 (5.77)	3.90 (2.53)	4.44 (2.82)	5.26 (3.90)	0.215
Clinical dementia rating, score, mean (SD)	0 to 3	1.29 (0.56)	1.39 (0.58)	1.36 (0.67)	1.22 (0.47)	1.24 (0.48)	0.520
CR functional impairment, score, %, mean (SD)	0 to 100	65.9 (18.4)	68.9 (21.6)	64.8 (19.6)	64.4 (17.4)	69.3 (15.2)	0.647
Perceived caregiver burden, score, mean (SD)	12 to 60	32.9 (7.6)	35.2 (7.3)	32.7 (7.1)	32.3 (8.7)	32.4 (5.9)	0.514
Caregiving-specific stress total, z-score, mean (SD)	−1.91 to 3.14	0.00 (0.66)	0.24 (0.85)	−0.04 (0.68)	−0.08 (0.57)	0.05 (0.57)	0.271

Significant differences between groups of SRH (*p* < 0.05): ^1)^ poor/fair SRH vs. excellent SRH; ^2)^ poor/fair SRH vs. very good SRH; ^3)^ poor/fair SRH vs. good SRH; ^4)^ good SRH vs. excellent SRH; ^5)^ good SRH vs. very good SRH; ^6)^ very good SRH vs. excellent SRH; CR = care recipient

There were however, significant differences in health behaviors, physical health indicators and affect between the four groups. Higher levels of physical health problems, lower caregiver physical function and more negative affect differentiated caregivers with poor/fair SRH from those with good SRH. In addition to these characteristics, less physical activity and less alcohol consumption differentiated caregivers with poor/fair SRH from those with very good and excellent SRH, respectively. Moreover, higher BMI and lower positive affect differentiated caregivers with poor/fair SRH from those with excellent SRH. Caregivers with good SRH also differed significantly in several health behaviors and physical health indicators from those with very good or excellent SRH. High negative affect and low positive affect differentiated caregivers with very good SRH from those with excellent SRH.

### Association of caregiving-specific stress with demographic and health characteristics

We first explored associations of measures of caregiving-specific stress with the other determinants of SRH. Such relationships may help to interpret potential differences in the link between caregiving-specific stress and SRH, which may emerge between the crude and fully-adjusted regression analyses presented below.

Higher total caregiving-specific stress was associated with younger age (r = − 0.21, *p* = 0.017), better physical function (r = 0.21, *p* = 0.014) and more negative affect (r = 0.23, *p* = 0.008). There emerged several significant associations of individual caregiving-specific stressors with demographic and health characteristics. Greater care recipient dementia was associated with higher BMI (r = 0.21, *p* = 0.013), less alcohol consumption (r = − 0.20, *p* = 0.022) and more social support (r = 0.22, *p* = 0.011). Greater care recipient functional impairment was associated with less alcohol consumption (r = − 0.26, *p* = 0.002) and better caregiver physical function (r = 0.21, p = 0.017). Higher perceived caregiver burden was associated with younger age (r = − 0.41, *p* < 0.001), male sex (r = 0.34, *p* < 0.001) and more negative affect (r = 0.48, *p* < 0.001).

### Determinants of poor/fair versus good, very good, or excellent self-rated health

Table 2 shows the crude and fully-adjusted likelihood for the association of demographic factors, health characteristics (health behaviors, physical health indicators, psychosocial factors) and caregiving-specific stress total with caregivers’ SRH status, with poor/fair SRH (Group 1 (G1) as the reference category compared to caregivers with good SRH (G2), very good SRH (G3) and excellent SRH (4). Significant crude relationships emerged for BMI (*p* = 0.013), physical activity (*p* < 0.001), alcohol consumption (*p* = 0.008), physical health problems (*p* < 0.001), caregiver physical function (p < 0.001), negative affect (*p* < 0.001) and positive affect (p < 0.001), but not for caregiving-specific stress total. Several differences regarding the likelihood of different health behaviors (i.e., BMI, physical activity, alcohol consumption) between G1 and G3 and between G1 and G4 were no longer significant after adjustment for covariates.

**Table 2:** Multinomial logistic regression analyses of determinants of self-rated health in 134 Alzheimer caregivers

Factors	Good SRH vs. poor/fair SRH		Very good SRH vs. poor/fair SRH		Excellent SRH vs. poor/fair SRH	
	Crude OR (95% CI)	Adjusted OR (95% CI)	Crude OR (95% CI)	Adjusted OR (95% CI)	Crude OR (95% CI)	Adjusted OR (95% CI)
Age	0.98 (0.92, 1.05)	1.01 (0.90, 1.13)	1.02 (0.96, 1.09)	**1.15 (1.02, 1.30)**	1.00 (0.93, 1.08)	1.09 (0.93, 1.28)
Female sex	0.61 (0.15, 2.55)	0.67 (0.09, 4.73)	0.50 (0.13, 1.98)	0.30 (0.04, 2.42)	0.63 (0.12, 3.24)	0.19 (0.01, 2.66)
Higher education	1.13 (0.35, 3.63)	0.91 (0.16, 5.14)	1.33 (0.42, 4.19)	0.81 (0.13, 5.09)	7.20 (0.78, 66.63)	3.56 (0.19, 66.64)
Body mass index	0.97 (0.90, 1.06)	1.08 (0.93, 1.26)	0.92 (0.83, 1.01)	1.16 (0.97, 1.39)	**0.81 (0.70, 0.94)**	0.96 (0.74, 1.25)
Physical activity	1.02 (0.71, 1.46)	0.88 (0.51, 1.53)	**1.48 (1.04, 2.10)**	1.20 (0.66, 2.17)	**2.02 (1.30, 3.14)**	1.52 (0.75, 3.10)
Alcohol consumption	1.13 (0.84, 1.51)	1.29 (0.80, 2.08)	**1.38 (1.05, 1.83)**	**1.88 (1.14, 3.12)**	**1.50 (1.09, 2.06)**	1.73 (0.96, 3.10)
Ever smoking	0.56 (0.19, 1.61)	1.08 (0.26, 4.49)	0.57 (0.20, 1.58)	0.89 (0.18, 4.33)	1.25 (0.36, 4.36)	**10.01 (1.10, 91.14)**
Physical health problems	0.77 (0.56, 1.05)	0.80 (0.51, 1.26)	**0.51 (0.35, 0.74)**	**0.55 (0.31, 0.97)**	**0.47 (0.28, 0.77)**	0.57 (0.26, 1.27)
Caregiver physical function	**1.56 (1.18, 2.10)**	**2.78 (1.49, 5.21)**	**2.09 (1.52, 2.88)**	**4.42 (2.21, 8.81)**	**2.81 (1.75, 4.52)**	**6.25 (2.75, 14.20)**
Negative affect	**0.86 (0.79, 0.93)**	**0.88 (0.78, 0.99)**	**0.91 (0.84, 0.98)**	0.97 (0.86, 1.09)	**0.81 (0.72, 0.91)**	**0.78 (0.64, 0.96)**
Positive affect	1.02 (0.95, 1.11)	1.08 (0.95, 1.22)	1.07 (0.99, 1.16)	**1.17 (1.02, 1.35)**	**1.22 (1.10, 1.36)**	**1.33 (1.11, 1.60)**
Social support	1.08 (0.98, 1.20)	1.03 (0.89, 1.20)	1.00 (0.91, 1.10)	0.91 (0.77, 1.08)	1.07 (0.95, 1.21)	1.01 (0.80, 1.26)
Caregiving-specific stress total	0.81 (0.62, 1.05)	**0.57 (0.37, 0.87)**	0.78 (0.61, 1.01)	**0.54 (0.35, 0.84)**	0.87 (0.64, 1.18)	**0.51 (0.30, 0.86)**

Odds ratio (OR) with 95% confidence interval (CI) in bold indicates a significant difference in the likelihood of a variable from the group of poor/fair self-rated health (SRH) as the reference category. All variables were entered in one block. Caregiver physical function and caregiving-specific stress total are expressed for half a standard deviation increase in the averaged z-score computed from the five, respectively four, original variables included in these scores

Range of scores: 0 to 6 for physical activity and for alcohol consumption, 0 to 8 for physical health problems, −5.54 to 2.12 for caregiver physical function, 10 to 40 for negative affect, 10 to 50 for positive affect, 1 to 24 for social support, −4.54 to 5.58 for caregiving-specific stress total

In the fully adjusted model, age (*p* = 0.006), alcohol consumption (*p* = 0.011), ever smoked (*p* = 0.022), caregiver physical function (*p* < 0.001), negative affect (*p* = 0.003), positive affect (*p* = 0.005), and caregiving-specific stress total (*p* = 0.027) made significant unique contributions. G1 showed a greater likelihood of lower caregiver physical function and higher caregiving-specific stress total compared to G2, G3 and G4. Moreover, in G1 the likelihood of negative affect was higher than in G2 and G4, whereas the likelihood of positive affect was lower in G1 than in G3 and in G4. Also, the likelihood of being younger, consuming less alcohol, and more physical health problems was higher in G1 than in G3. Rather unexpectedly, the likelihood of ever smoking was lower in G1 than in G4.

Post hoc analyses on individual caregiving-specific stressors are shown in Table 3. Caregivers in G1 were more likely to have provided care for more years than those in G2, G3 and G4. Also, there was a greater likelihood of more severe dementia of the care recipient in G1 than in G3 and G4, and of greater care recipient functional impairment in G1 than in G2.

**Table 3:** Adjusted likelihood of caregiving stressors predicting self-rated health

Factors	Good SRH vs. poor/fair SRH	Very good SRH vs. poor/fair SRH	Excellent SRH vs. poor/fair SRH
	OR (95% CI)	OR (95% CI)	OR (95% CI)
Years of caregiving	**0.11 (0.02, 0,59)**	**0.16 (0.03, 0.73)**	**0.04 (0.01, 0.34)**
Clinical dementia rating	0.37 (0.10, 1.43)	**0.23 (0.05, 0.99)**	**0.13 (0.02, 0.98)**
Care recipient functional impairment	**0.16 (0.03, 0.97)**	0.16 (0.03, 1.07)	0.21 (0.02, 2.06)
Perceived caregiver burden	0.65 (0.10, 4.35)	0.52 (0.07, 3.89)	1.71 (0.12, 24.25)

Multinomial logistic regression models were adjusted for age, sex, education, body mass index, physical activity, ever smoking, alcohol consumption, physical health problems, caregiver physical function, negative affect, positive affect, and social support. An odds ratio (OR) with 95% confidence interval (CI) in bold indicates a significant difference in the likelihood of half a standard deviation increase in standardized z-scores of a caregiving-specific stressor variable from the group of poor/fair self-rated health (SRH) as the reference category

Range of scores: −0.59 to 2.60 for years of caregiving, −0.71 to 1.53 for clinical dementia rating, −1.34 to 0.89 for care recipient functional impairment, −1.17 to 1.25 for perceived caregiver burden

### Complementary analyses with self-rated health as a binary variable

Due to the small sample size in the group of caregivers with excellent SRH, there were wide confidence intervals for “higher education” and “ever smoking” as determinants of excellent SRH vs. poor/fair SRH. Therefore, we performed complementary analyses with SRH as a binary outcome (Additional file 1: Tables S1 and S2). In fully adjusted logistic regression post hoc analyses, lower caregiver physical function (*p* < 0.001), more negative affect (*p* = 0.029) and more caregiving-specific stress total (*p* = 0.005), differentiated caregivers with poor/fair SRH from those with either good, very good or excellent SRH. Of the individual caregiving-specific stressors, more years of caregiving (*p* = 0.006) and greater care recipient functional impairment (*p* = 0.049) were significantly higher in caregivers with poor/fair SRH.

## Discussion

The objective of this study was to identify determinants of poor/fair SRH versus self-assessments of good, very good or excellent health in a sample of informal caregivers providing in-home care for a spouse with dementia. Of 134 caregivers studied, 15.7% rated their own health as poor or fair. This proportion is lower than in earlier studies with percentages ranging between 18 and 45% and a median of 31% [23], but closer to more recent surveys in which 20% of caregivers described their own health as poor or fair [15]. In turn, the high proportion of caregivers describing their health as good, very good or excellent could also reflect evidence of caregiver resilience, even among spousal dementia caregivers who are a particularly vulnerable subgroup.

Both in the crude and fully adjusted analysis, we found that caregivers who reported poor/fair SRH had lower physical function. The likelihood of higher caregiver physical function increased from good to very good to excellent SRH in the fully adjusted model. Medical comorbidity, defined by the number of physical health problems, was a less robust predictor and more likely in caregivers with poor/fair vs. those with very good SRH only. Therefore, reports of any impairment in caregiver physical function may be more relevant to SRH than the sheer number of physical health problems.

The probability of greater total caregiving-specific stress was also higher in caregivers with poor/fair SRH, as opposed to caregivers in the other three categories of SRH. However, this was true only after controlling for covariates, likely because caregiver physical function and total caregiving-specific stress were directly correlated with each other. In other words, only when their mutual effect was accounted for was caregiving-specific stress total unmasked as an independent determinant of poor/fair SRH.

Caregivers with higher physical function may be those having more caregiving-specific stress because their physical resources make them more capable of taking care of an overly ill and impaired spouse. This is underscored by the observation that higher caregiver physical function was also correlated with greater care recipient functional impairment. In the post hoc analysis, a higher probability of measures of objective caregiving-specific stressors – more years of caregiving, more severe dementia and greater care recipient functional impairment distinguished poor/fair SRH from categories of at least good SRH.

Contrary to a study from Iran in which perceived caregiver burden emerged as the strongest predictor of SRH, perceived caregiver burden was not associated with SRH in our sample [25]. Different measures of perceived caregiver burden and SRH, cultural differences in caregiver roles, and, in the study on dementia caregivers from Iran, a high proportion of non-spousal family caregivers (two thirds were daughters/sons) and a comparably lower socioeconomic status (two fifths being poor or very poor) might possibly explain this inconsistency. A longer duration of caregiving was most consistently associated with a higher probability of poor/fair SRH; in agreement with data showing that 17% of caregivers believe that their health has deteriorated as a result of providing care, particularly those who have been providing care for at least 5 years [15].

The likelihood of more negative affect and less positive affect was also highest in caregivers with poor/fair SRH. The finding regarding negative affect concurs with literature showing that poor SRH is associated with psychological distress and psychiatric morbidity, including depressive symptoms in dementia caregivers [23, 26]. The role of positive affect in SRH has to our knowledge not previously been investigated in caregivers. More negative affect already distinguished caregivers with poor/fair SRH from those with good SRH, whereas more positive affect was likeliest in those with very good and excellent vs. poor/fair SRH.

A direct comparison between categories of SRH showed more positive affect in those with excellent vs. very good or good SRH (Table 1). In older people, followed for up to five years, positive affect, as opposed to negative affect, was associated with changes in SRH only when physical health (chronic disease score) was better than usual [39]. Another study in community-dwelling older adults showed that participants with higher positive affect were more likely to preserve favorable assessments of SRH over a follow-up between one and five years, even after adjustment for functional limitations [40]. Altogether, high positive affect may be necessary for dementia caregivers to perceive their health at the very positive end. Moreover, for a better understanding of the basis of SRH in dementia caregivers, positive affect measures should be included in future studies, along with negative affect and medical comorbidity, to attend to the full illness-wellness continuum of health and not only to the presence or absence of disease [40].

Of interest, there was little evidence for differences in demographic factors and adverse health behaviors between caregivers with poor/fair vs. groups with at least good SRH when controlling for covariates. This is in agreement with a study showing that socioeconomic and health behavior covariates predicted mortality in individuals with average and good SRH, but not in those with poor SRH, whose risk was largely attributable to illness-related limitations [41]. Exceptions in our sample were lower alcohol consumption, concurring with some positive health effects of moderate alcohol intake in older adults [42], and younger age, both in caregivers with poor/fair vs. those with very good SRH. Younger caregivers could be more concerned than older ones that caregiving adversely impacts their own health. We believe the unexpected finding of a higher probability of ever smokers in caregivers with excellent vs. poor/fair SRH is due partially to the fact that among the 58 participants identifying as “ever smokers”, only 5 reported they were “current” smokers, meaning that over 90% had quit smoking at some point in their lifetimes. Further, it is possible that among the remaining “current” smokers, smoking is used as a stress relief, which has been demonstrated in the scientific literature [43].

On the whole, the findings from this study suggest that poor/fair SRH vs. at least good SRH reflects an inclusive measure of low physical and mental health as well as caregiving-specific stress in dementia caregivers. These domains have individually been shown to be associated with physical health decline and mortality in caregivers [1, 7, 11, 12, 44]. Therefore, from a clinical perspective, screening for poor/fair SRH with a single-item question may be valuable for identifying caregivers at risk of adverse health outcomes [15, 17]. Even in a busy clinical setting, such a policy could be applied to capture a need for further clinical evaluation and specific treatments. A better understanding of correlates of poor/fair SRH may prompt target interventions aimed at improving SRH in the most vulnerable caregivers. When appropriate treatment follows positive screening, a caregiver’s perceived poor or fair health could be improved. Meta-analyses show that interventions improve caregiver burden and negative emotions, but less so physical health [45, 46]. Yet, compared with a no-treatment control group, dementia caregivers who underwent a 6-month multicomponent skills training, targeting self-care behaviors, showed improvements in physical health, mood, and SRH assessed with a single-item question [47]. With periodical assessments of SRH, clinicians could also easily monitor treatment success and sustainability. However, whether this clinical approach ultimately results in better physical health outcomes and lower mortality of informal caregivers remains to be demonstrated. Unfortunately, as the intervention study from which we drew data for this paper was not designed to specifically improve SRH, post-intervention data were not included in this analysis.

Limitations of our study are the cross-sectional design, precluding any causal inferences, and a modest percentage of caregivers with poor/fair SRH limiting statistical power. This may be a consequence of the aim of our parent study and may threaten external validity of our findings. Caregivers were originally enrolled in an intervention trial, so they may have felt overly healthy to even dare taking the effort to participate; moreover, we excluded caregivers with severe medical conditions. Nevertheless, all caregivers were at least mildly distressed per enrollment criteria, so they reflect an important subgroup of this vulnerable population. The high proportion of female caregivers and an above-average socioeconomic status, with only two caregivers indicating less than grade 12 education, are possible reasons for why sex and education were not predictive of poor/fair SRH.

In terms of representativeness, our sample is highly consistent with epidemiologic samples of spousal dementia caregivers in the United States, and in California in particular, as seen by the statistics provided by the Alzheimer’s Association in their annual “Facts and Figures” release [48]. Indeed we have a majority of our sample as female, with approximately 40% having significant symptoms of depression, and providing substantial amounts of care per week (both in terms of hours caregiving and services provided). However, because we did not have specific resources available to enroll Spanish-speaking participants, our sample does have potential bias in terms of race/ethnicity, limiting its representativeness. Caution is advised when generalizing our findings to samples with a higher share of male caregivers and caregivers without formal education. We used a systems review and history questionnaire to assess medical comorbidity, but there are more established measures such as the Charlson comorbidity index. There are various response formats to assess SRH [21] and some do not apply single-item questions [23], making an integration of the literature challenging. As they are not part of clinical routine, we did not consider biomarkers of disease risk, which are elevated in dementia caregivers [5] and also associated with SRH [49]. However, SRH has been shown to predict mortality even when controlling for objectively confirmed laboratory data [21]. We acknowledge that estimates for a few determinants of excellent SRH are uncertain due to the small number of caregivers in this category. Nonetheless, informative knowledge that higher levels of positive affect and less severe dementia in the care recipient may differentiate caregivers with different SRH response categories from those with poor/fair SRH was lost in the binary logistic analysis. As the relationship between dementia caregiving and different categories of SRH is still a nascent field of research, we decided to present and discuss the findings from the multinomial logistic regression analysis as the primary results of our study.

## Conclusions

Taken together, dementia caregivers with poor/fair SRH are more likely to have medical comorbidity, low physical function, high negative affect, low positive affect, and more subjective caregiving-specific stressors than caregivers who self-rate their health as good, very good or excellent. A single-question rating of self-perceived health may be a clinically useful tool to evaluate caregivers’ physical health risk in clinical practice and a need for further clinical evaluation and treatment.

## Additional file

Additional file 1: **Table S1.** Binary logistic regression analysis of determinants of self-rated health in 134 Alzheimer caregivers. The Table shows the fully adjusted differences in demographic factors, health behaviors, physical health indicators, psychosocial factors and caregiving-specific stressors between the group of caregivers with either good, very good or excellent self-rated health (*n* = 113) and the group of caregivers with either poor or fair self-rated health (*n* = 21). **Table S2.** Adjusted likelihood of caregiving stressors predicting self-rated health. The Table shows the fully adjusted differences in caregiver stressors between the group of caregivers with either good, very good or excellent self-rated health (*n* = 113) and the group of caregivers with either poor or fair self-rated health (*n* = 21). (ZIP 19 kb)

## Abbreviations

BMI: Body mass index
G: Group
SRH: Self-rated health
UCSD: University of California San Diego
