# Supplementary material for: Refining caregiver vulnerability for clinical practice: determinants of self-rated health in spousal dementia caregivers
Source: BMC Geriatr. 2019 Jan 22;19:18. doi: 10.1186/s12877-019-1033-2 (PMC6343283; doi:10.1186/s12877-019-1033-2)
Supplement: Supplementary file 1 — Table S1. Binary logistic regression analysis of determinants of self-rated health in 134 Alzheimer caregivers. The Table shows the fully adjusted differences in demographic factors, health behaviors, physical health indicators, psychosocial factors and caregiving-specific stressors between the group of caregivers with either good, very good or excellent self-rated health (n = 113) and the group of caregivers with either poor or fair self-rated health (n = 21). Table S2. Adjusted likelihood of caregiving stressors predicting self-rated health. The Table shows the fully adjusted differences in caregiver stressors between the group of caregivers with either good, very good or excellent self-rated health (n = 113) and the group of caregivers with either poor or fair self-rated health (n = 21). (ZIP 19 kb) [file 12877_2019_1033_MOESM1_ESM.zip › Additional File 1_Table S1R3.docx]

**Table S1. Binary logistic regression analysis of determinants of self-rated health in 134 Alzheimer caregivers**

| Factors | Good/very good/excellent SRH vs. poor/fair SRH | |
| --- | --- | --- |
|  | Crude OR (95% CI) | Adjusted OR (95% CI) |
| Age | 1.00 (0.95, 1.06) | 1.06 (0.96, 1.17) |
| Female sex | 0.56 (0.15, 2.04) | 0.59 (0.10, 3.63) |
| Higher education | 1.48 (0.52, 4.23) | 0.93 (0.19, 4.56) |
| Body mass index | 0.93 (0.87, 1.01) | 1.13 (0.98, 1.30) |
| Physical activity | 1.33 (0.98, 1.82) | 0.98 (0.58, 1.67) |
| Alcohol consumption | **1.31 (1.00, 1.70)** | 1.44 (0.91, 2.25) |
| Ever smoking | 0.65 (0.25, 1.65) | 1.12 (0.29, 4.35) |
| Physical health problems | **0.63 (0.47, 0.84)** | 0.70 (0.46, 1.09) |
| Care recipient physical function | **1.85 (1.41, 2.43)** | **3.28 (1.80, 6.00)** |
| Negative affect | **0.88 (0.82, 0.94)** | **0.89 (0.80, 0.99)** |
| Positive affect | 1.07 (0.98, 1.14) | 1.10 (0.98, 1.24) |
| Social support | 1.04 (0.96, 1.14) | 0.99 (0.86, 1.13) |
| Caregiving-specific stress total | 0.81 (0.64, 1.02) | **0.57 (0.38, 0.85)** |

Odds ratio (OR) with 95% confidence interval (CI) in bold indicates a significant difference in the likelihood of a variable from the group of poor/fair self-rated health (SRH) as the reference category. All variables were entered in one block. Caregiver physical function and caregiving-specific stress total are expressed for half a standard deviation increase in the averaged z-score computed from the five, respectively four, original variables included in these scores. Cf. legend Table 2 for range of scores
