# Supplementary material for: Refining caregiver vulnerability for clinical practice: determinants of self-rated health in spousal dementia caregivers
Source: BMC Geriatr. 2019 Jan 22;19:18. doi: 10.1186/s12877-019-1033-2 (PMC6343283; doi:10.1186/s12877-019-1033-2)
Supplement: Supplementary file 1 — Table S1. Binary logistic regression analysis of determinants of self-rated health in 134 Alzheimer caregivers. The Table shows the fully adjusted differences in demographic factors, health behaviors, physical health indicators, psychosocial factors and caregiving-specific stressors between the group of caregivers with either good, very good or excellent self-rated health (n = 113) and the group of caregivers with either poor or fair self-rated health (n = 21). Table S2. Adjusted likelihood of caregiving stressors predicting self-rated health. The Table shows the fully adjusted differences in caregiver stressors between the group of caregivers with either good, very good or excellent self-rated health (n = 113) and the group of caregivers with either poor or fair self-rated health (n = 21). (ZIP 19 kb) [file 12877_2019_1033_MOESM1_ESM.zip › Additional File 1_Table S2R3.docx]

**Table S2. Adjusted likelihood of caregiving stressors predicting self-rated health**

| Factors | Good/very good/excellent SRH vs. poor/fair SRH |
| --- | --- |
|  | OR (95% CI) |
| Years of caregiving | **0.14 (0.03, 0.58)** |
| Clinical dementia rating | 0.30 (0.08, 1.12) |
| Care recipient functional impairment | **0.17 (0.03, 0.99)** |
| Perceived caregiver burden | 0.52 (0.09, 3.24) |

Binary logistic regression models were adjusted for age, sex, education, body mass index, physical activity, ever smoking, alcohol consumption, physical health problems, caregiver physical function, negative affect, positive affect, and social support. An odds ratio (OR) with 95% confidence interval (CI) in bold indicates a significant difference in the likelihood of half a standard deviation increase in standardized z-scores of a caregiving-specific stressor variable from the group of poor/fair self-rated health (SRH) as the reference category. Cf. legend to Table 3 for range of scores.
